# The supramolecular structure and van der Waals interactions affect the electronic structure of ferrocenyl-alkanethiolate SAMs on gold and silver electrodes†

**DOI:** 10.1039/c9na00107g

**Published:** 2019-03-29

**Authors:** Liang Cao, Li Yuan, Ming Yang, Nisachol Nerngchamnong, Damien Thompson, Xiaojiang Yu, Dong-Chen Qi, Christian A. Nijhuis

**Affiliations:** Department of Chemistry, National University of Singapore 3 Science Drive 3 Singapore 117543 Singapore chmnca@nus.edu.sg; Anhui Province Key Laboratory of Condensed Matter Physics at Extreme Conditions, High Magnetic Field Laboratory of the Chinese Academy of Sciences 350 Shushanhu Road Hefei 230031 China; Department of Physics, National University of Singapore 2 Science Drive 3 Singapore 117542 Singapore; Institute of Materials Research and Engineering (IMRE), Innovis 2 Fusionopolis Way Singapore 138634 Singapore; Department of Physics, Bernal Institute, University of Limerick V94 T9PX Ireland; Singapore Synchrotron Light Source, National University of Singapore 5 Research Link Singapore 117603 Singapore; School of Chemistry, Physics and Mechanical Engineering, Queensland University of Technology Brisbane Queensland 4001 Australia dongchen.qi@qut.edu.au; Department of Chemistry and Physics, La Trobe Institute for Molecular Science, La Trobe University Melbourne Victoria 3086 Australia; Centre for Advanced 2D Materials and Graphene Research Centre, National University of Singapore 6 Science Drive 2 Singapore 117546 Singapore; NUSNNI-Nanocore, National University of Singapore Singapore 117411 Singapore

## Abstract

Understanding the influence of structural properties on the electronic structure will pave the way for optimization of charge transport properties of SAM devices. In this study, we systematically investigate the supramolecular and electronic structures of ferrocene (Fc) terminated alkanethiolate (SC_*n*_Fc) SAMs on both Au and Ag substrates with *n* = 1–15 by using a combination of synchrotron based near edge X-ray absorption spectroscopy (NEXAFS), photoemission spectroscopy (PES), and density functional theory (DFT) calculations. Odd–even effects in the supramolecular structure persist over the entire range of *n* = 1–15, which, in turn, explain the odd–even effects in the onset energy of the highest occupied molecular (HOMO) orbital. The orientation of the Fc moieties and the strength of Fc-substrate coupling, which both depend on *n*, affects the work function (WF). The variation of WF shows an odd–even effect in the weak electrode–Fc coupling regime for *n* ≥ 8, whereas the odd–even effect diminishes for *n* < 8 due to hybridization between Fc and the electrode (*n* < 3) or van der Waals (vdW) interactions between Fc and the electrode (*n* = 3–7). These results confirm that subtle changes in the supramolecular structure of the SAMs cause significant electronic changes that have a large influence on device properties.

## Abstract 2

Understanding the influence of structural properties on the electronic structure will pave the way for optimization of charge transport properties of SAM devices.

## Graphical abstract

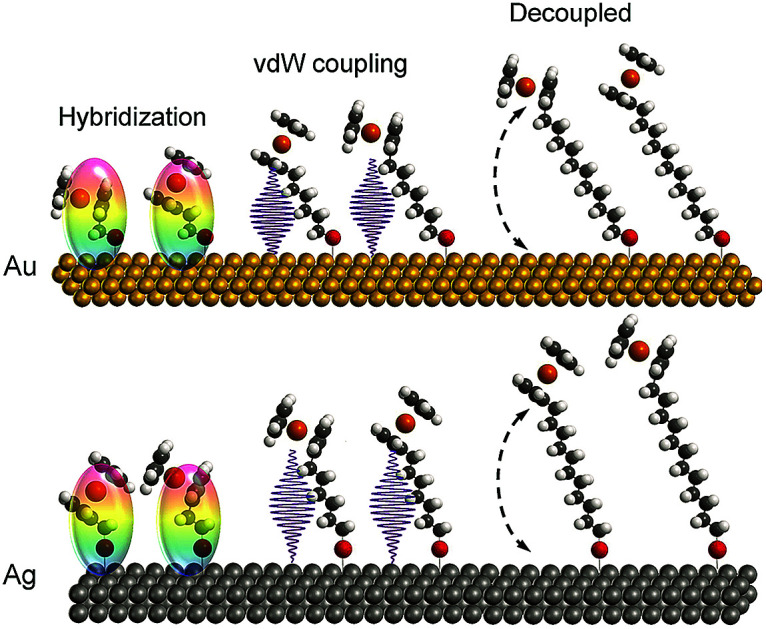

## Introduction

Self-assembled monolayers (SAMs) have been used to tailor the properties of metal surfaces in applications ranging from tribology,^1–3^ molecular and organic electronics,^4,5^ green energy,^6^ to nanofabrication.^7^ This is mainly because SAMs provide a convenient strategy to control the physical, chemical, and electronic properties of surfaces of metals and inorganic semiconductors.^7^ For applications in electronics, SAMs have been used to lower charge injection barriers,^8^ to improve packing of polymers and small molecules in organic thin film transistors,^9,10^ and as the active component in SAM-based molecular electronics.^7,11–16^ One of the advantages of SAMs in these applications is that they can be chemically tailored, allowing for atomic scale control over the electronic structure of the molecule–electrode interfaces, which, in turn, determines the performance of the SAM-based devices.^17–23^

SAMs of alkanethiolates on metals (*e.g.*, Au and Ag) are extensively studied because they form densely packed and well-ordered two-dimensional (2D) films.^7^ In these systems, SAM molecules are chemically anchored to the substrates *via* a metal–thiolate bond, and active (or functional) groups are (usually) located at the other end of a spacer moiety (often an alkyl chain or conjugated backbone). Redox-active functional groups provide low-energy molecular states close to the Fermi level (*E*_F_) of the electrode, *i.e.*, both occupied and unoccupied molecular orbitals, providing electronic functions that are useful for applications in molecular diodes, switches, spintronics, and opto-electronics,^24–29^ which can be integrated into novel molecular nanoelectronic devices.^19,30–32^ For example, we have previously reported that redox active ferrocenyl (Fc) terminated alkanethiolate SAMs can rectify currents with rectification ratios (*R*) of up to 6.3 × 10^5^.^33^ In principle, the electronic properties of electrode–SAM interfaces are tunable by chemical modification of the SAM precursors, but the interfacial energetics (*i.e.*, the energy level alignment) also depends strongly on the supramolecular structure of the SAM and how the molecules of the SAM interact with the electrode.^34–41^ The difference between *E*_F_ of the electrode and the energy of the frontier highest occupied molecular orbital (HOMO), *E*_HOMO_, or lowest unoccupied molecular orbital (LUMO), *E*_LUMO_, levels of SAMs is often related to the current injection efficiencies, turn-on values of switches and diodes, Fermi-level pinning and surface dipoles.^40,42–44^

Active groups such as ferrocenes add functionality to the SAMs, but they usually have larger diameters than the alkyl chain spacer and, as a consequence, they affect the packing structure of the SAM.^7,19^ Hence, these SAMs provide a good opportunity to study the interplay between electronic and supramolecular structures at SAM–metal interfaces.^45–52^ One way to study this interplay is to probe odd–even effects where the number, *n*, of a small repeat unit (here a methylene CH_2_ unit of the alkyl chain backbone of the SAM) is systematically changed and SAMs with an odd number of repeat units have different properties than SAMs with an even number of repeat units.^53^ For large values of *n* one would expect the functional group to be completely decoupled from the electrode and changes in *n* would primarily affect the supramolecular structure of the SAM. For SAMs with *n* approaching 0, one would expect that the functional group hybridizes with the bottom electrode *via* electron delocalization across the sulfur anchoring group which would alter the energy level alignment of the system.^17^ For intermediate values of *n*, we have previously shown that van der Waals coupling of the functional group and the electrode can be important.^17^ However, the value of *n* at which changes in electronic structure dominate over those in the supramolecular structure is usually poorly defined and unknown for most systems.

**Fig. 1:**
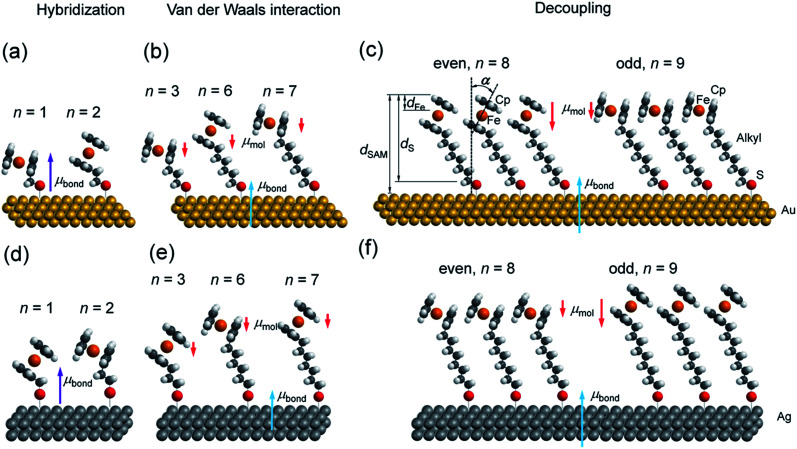
Schematic view of representative SC_*n*_Fc SAMs on (a–c) Au and (d–f) Ag substrates. Hybridization region is represented for *n* < 3; vdW interaction region is represented for 3 ≤ *n* ≤ 7; decoupled region is shown for larger *n*. Induced bond dipoles and molecular dipoles are discussed in the text. The blue arrows indicate *μ*_bond_ associated with the M–S bond, the purple arrows indicate *μ*_bond_ associated with both the M–S bond and hybridization of the Fc unit with the surface, and the red arrows indicate *μ*_mol_ along the alkyl chain.

We have reported odd–even effects in the tunneling rates across molecular diodes of the form Ag–SAM/EGaIn with SC_*n*_Fc SAMs.^54^ Here, junctions with an odd numbered *n*, SAM_odd_, performed better than those junctions with an even numbered *n*, SAM_even_, over a range of values of *n* = 6–15, while the odd–even effects were reversed for junctions with Au electrodes.^19^ We have also reported an odd–even effect on the charge transfer rates across these SAMs on Au for *n* = 0–15 under wet electrochemical conditions,^55^ which were confirmed by others.^45^ The odd–even effect in the supramolecular structure of the SAM was also observed with scanning tunneling microscopy, but only for SAMs on Au with *n* = 3 and 4.^56^ Here we wish to detail the supramolecular and electronic structure of these SAMs, on both template-stripped Au (Au^TS^) and Ag (Ag^TS^) films, by extending the range of *n* values from 6–15 to 1–15. This paper builds on our previous work^54,55^ in order to address in detail the following two questions: (i) how does the length (*n*) of the alkyl backbone of the SAM, which acts as insulator and decouples the substrate and Fc groups, affect the supramolecular structure of SAMs (illustrated in Fig. 1)? (ii) How does the supramolecular structure of the SAMs, in turn, alter the electronic structure of the SAM–electrode interface?

Synchrotron-based near edge X-ray absorption fine structure (NEXAFS), photoemission spectroscopy (PES), and atomic-scale molecular dynamics (MD) and density functional theory (DFT) calculations were employed to examine the evolution of packing and electronic structures of the SAMs as a function of *n*. Here we report the following four findings. (i) Reversal of the odd–even effects on the average tilt angles of the Fc moieties for SAMs on Au and SAMs on Ag persists over the entire range of values of *n* of 1–15. For all values of *n*, the average tilt angles of the Fc moieties in SAM_odd_ is larger than that of SAM_even_ on the Au surfaces, whereas this behaviour is reversed on Ag surfaces (*cf.*Fig. 1). This difference in behaviour can be explained by the difference in metal–S–C binding geometry (Ag–S–C and Au–S–C bond angles are close to 180° and 109° (ref. 53)) and proves that the odd–even effects for all values of *n* are driven by molecular packing. (ii) Odd–even effects in the electronic structure of the SAMs with *n* > 8 are only caused by the supramolecular structure of the SAMs, which can be explained by the change in molecular orientation and surface dipole. (iii) At intermediate values of 3 < *n* < 8, the energy level alignment of the system depends on *n* due to van der Waals coupling of the Fc units with the Au or Ag electrode. (iv) The electronic structure of SAMs with *n* < 3 is dominated by the hybridization between the Fc unit and the substrate across the *n* CH_2_ units and the Au–S bond.

## Experimental

Detailed synthetic procedures and characterization of HSC_*n*_Fc with 1 ≤ *n* < 6 can be found in ref. 55, whereas those for longer chain lengths of 6 ≤ *n* ≤ 15 are given in ref. 19. We formed the SAMs on ∼500 nm Au or Ag surfaces obtained by template-stripping (TS) following the same procedure as described in ref. 57. Briefly, cleaned glass slides (0.5 × 1.0 cm^2^) were employed to template strip as-deposited Au or Ag films from the SiO_2_/Si wafer by using optical adhesive (Norland, No. 61). The root mean square (rms) surface roughness of the TS Au (Au^TS^) was 0.5 nm, and that of TS Ag (Ag^TS^) 0.8 nm, both measured by atomic force microscopy (AFM) over an area of 1 × 1 μm^2^.^58,59^ SAMs were formed by immersing freshly prepared Au^TS^ or Ag^TS^ films in ∼3 mM solutions of HSC_*n*_Fc in ethanol under an atmosphere of N_2_ for 3 h at room temperature followed by rinsing with pure ethanol. After blowing the samples to dryness under a stream of N_2_, they were transferred into an ultrahigh vacuum (UHV) chamber (1 × 10^−10^ mbar) for synchrotron characterization.

The synchrotron-based NEXAFS and PES measurements were performed at the Surface, Interface, and Nanostructure Science (SINS) beamline of Singapore Synchrotron Light Source equipped with a Scienta R4000 electron energy analyzer following previously reported methods.^60^ All measurements were performed at room temperature. The photon energy was calibrated using the Au 4f_7/2_ core level peak at 84.0 eV of a sputter-cleaned gold foil in electrical contact with the sample. The spectra collected at different incident angles or electron take-off angles were obtained by rotating the sample stage. The C K-edge NEXAFS spectra were collected in Auger electron yield (AEY) mode by employing the electron analyzer, yielding a higher surface sensitivity than other collection modes (*e.g.*, total electron yield or partial electron yield). The linear polarization factor of the X-ray beam was 90%. All NEXAFS spectra were first normalized to the incident photon intensity (*I*_0_) monitored by the photocurrent of the refocusing mirror. Furthermore, the spectra were normalized by an *I*_0_ corrected NEXAFS spectrum monitored using the AEY mode on freshly sputtering cleaned Au foil. This double-normalization procedure ensures that the absorption features introduced by the carbon contamination on the beamline optics can be accounted for. Finally, the spectra were normalized to have the same edge jump between 280 eV and 320 eV in order to derive the angular dependence. A photon energy of 60 eV was employed to probe the valence band spectra. The binding energy (BE) is determined relative to the *E*_F_ of the sputter-cleaned gold foil. All the PES spectra were normalized by the photon flux. The work function (WF) was measured using 60 eV photon energy with −10 V bias applied to the sample.

All electronic structure calculations of the thiolate Au–SC_1_Fc and Au–SC_5_Fc complexes were carried out using density-functional theory (DFT) performed with the Vienna ab initio simulation package (VASP).^61^ The procedure is described in detailed in ref. 62. Briefly, The Heyd–Scuseria–Ernzerhof (HSE06) hybrid functional was used for the exchange correlation functional.^63^ The projector-augmented-wave (PAW) pseudopotentials, as implemented in VASP, were used for the interaction between electrons and ions.^64^ The cut-off energy of 400 eV was used for the plane-wave expansion of electron wave function. Vacuum regions larger than 10 Å were applied along all directions of the molecule to minimize the interaction with its nearest images. A 1 × 1 × 1 *Γ*-point-centered *k*-point mesh was used, and atomic coordinates in the structure were fully optimized until the force on each atom was smaller than 0.02 eV Å^−1^.

The molecular dynamics (MD) simulations based on parameters derived from DFT models, are described in ref. 19. Briefly, the structure was calculated by using MD simulations from statistically 1216 ferrocenyl-alkanethiol molecules adsorbed on Au(111) or Ag(111) surface with areas of 33 × 13 nm^2^. Molecular Langevin dynamics were performed using the NAMD program.^65^ Each model was encased in a large vacuum box of edge length 50 nm and periodic boundary conditions were applied. Ewald summation was used to calculate the electrostatic interactions and a 2 fs time-step was used for dynamics by constraining covalent bonds to hydrogen. The substrate Ag atoms were restrained to their crystallographic positions throughout the simulations. Each model was first relaxed using 2000 steps of steepest descent minimization with respect to the CHARMM22 force field^66^ and then brought to room temperature by gradually raising the temperature from 0 to 295 K over 2 ns of dynamics while simultaneously loosening positional constraints on the molecule non-hydrogen atoms. Each SAM (∼80 000 atoms) was allowed to equilibrate to a stable room temperature structure over 2 ns of MD and then subjected to a further 15 ns of dynamics. In every case, a constant-density monolayer formed within 5 ns. A weak additional non-bonded potential was introduced between chain sulphur atoms and the substrate, to model monolayer formation during physisorption to metal (*i.e.*, weak, non-specific SAM to substrate bonds). Following 10 ns of room temperature dynamics, individual Ag–S bonds were introduced into the SAM with a target Ag–S–C bond angle of 180°.^53^ For calculations on Au(111) we set a target Au–S–C bond angle of 109°.^66^ To minimise edge effects, metal–S bond formation and subsequent analysis was restricted to within a central disk of 5 nm radius (∼250 molecules). Each chemisorbed SAM was then sampled for an additional 5 ns. Statistics were generated from the final 2 ns of dynamics for each model, sampling every 20 ps to provide 100 statistically independent structures for each SAM. All data points and error bars reported were calculated over approximately 25 000 molecule conformations for each SAM (a central ∼250-molecule sampling disk sampled 100 times). Image generation and Tcl script-based trajectory analysis were performed using the VMD program.^67^

## Results and discussion

### Interpretation of the NEXAFS spectra

**Fig. 2:**
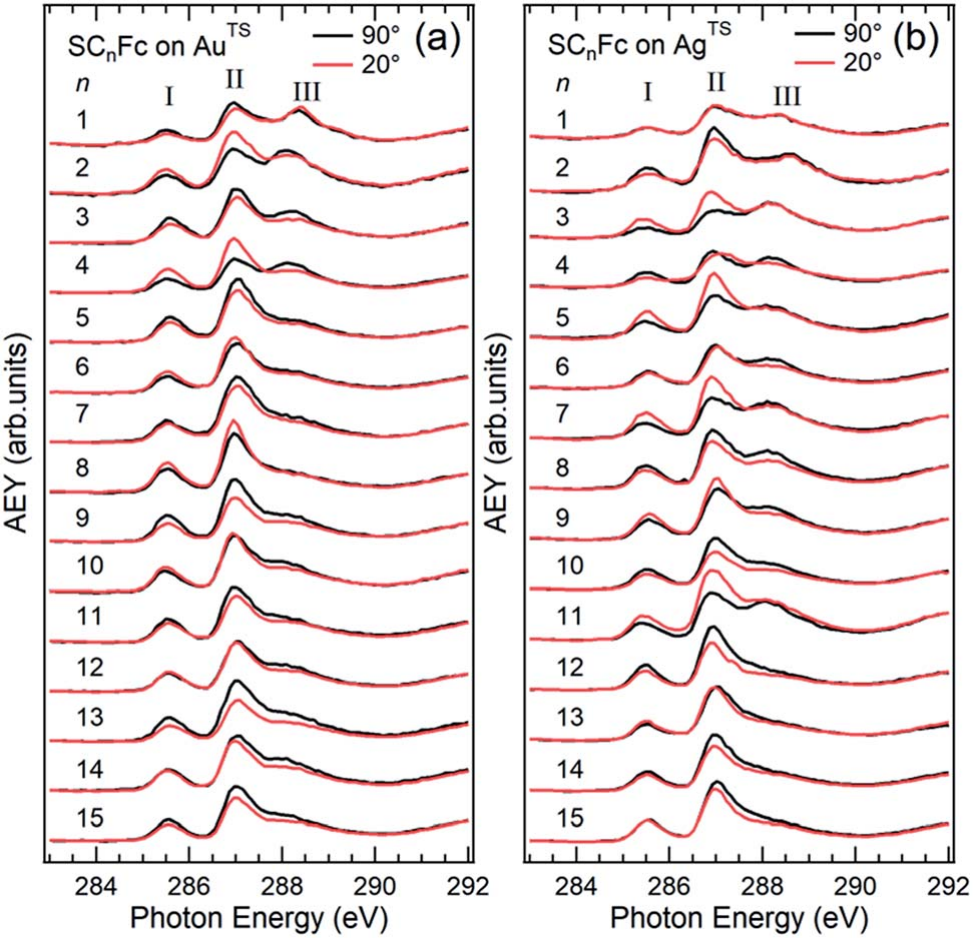
Angular dependent C K-edge NEXAFS spectra recorded on SC_*n*_Fc SAMs on (a) Au^TS^ and (b) Ag^TS^ surface at normal (*θ* = 90°) and grazing (*θ* = 20°) incidence. The X-ray incident angles *θ* are referred to the substrate surface. Data for *n* = 3 and 4 for SAMs on Au were taken from ref. 56. Data for *n* = 6–15 were taken from ESI of ref. 19. All data are shown together for the sake of clarity.

The Ag^TS^ and Au^TS^ surfaces used in this study as SAM substrates have a very low surface roughness^58,59^ and can be template-stripped on demand to minimize potential contamination from the environment.^57^ The Fc moiety consists of two cyclopentadienyl rings (Cp) and Fe(ii) as shown in Fig. 1. Fig. 2 shows the angular dependent NEXAFS at the C K-edge for SC_*n*_Fc SAMs on both Au^TS^ and on Ag^TS^ for *n* = 1–15. The experimental spectra are in good agreement with previously reported NEXAFS spectra obtained for S(CH_2_)_11_Fc SAMs on Au.^68^ The well-defined peaks labeled as I, II, and III, below 290 eV are attributed to the electronic transitions from C 1s to individual π* and C–S orbitals, respectively. The broad signals at photon energies above 290 eV are attributed to other σ* transitions (see Fig. S1† for spectra with photon energies up to 320 eV). The peak I occurring at ∼285.5 eV is attributed to transitions to π*(Cp) molecular orbitals (MOs) strongly mixed with Fe 3d_*xz*_ and 3d_*yz*_ contributions. The strong resonant peak II corresponds to the transitions to π*(Cp) orbitals weakly mixed with Fe 3d_*xy*_ and 3d_*x*^2^–*y*^2^_ contributions.^68–71^ Peak II is more delocalized than peak I as revealed by charge transfer dynamics results and DFT calculations due to much more localized character of the Fe 3d orbitals.^62^ This peak had been formerly assigned to π*(Cp) orbital without Fe 3d character.^70,72^ Otero *et al.*,^69^ however, found that the Fe 3d orbitals contribute to this state as well, which was confirmed by our own calculations in ref. 62. It is worth noting that both the peak positions and the relative intensities of peaks I and II do not show significant chain-length dependence, suggesting that the *E*_LUMO_ associated with the Fc moiety does not significantly change even when the Fc is strongly interacting with the substrate when *n* < 3 (below we show that the opposite is true for the HOMO states in agreement with ref. 17).

The additional peak III located at ∼288 eV, which was not observed from the NEXAFS spectrum of free ferrocene molecules,^69^ was previously assigned to C 1s (–CH_2_–) → σ* (C–H)/Rydberg transition,^68^ but we believe it originates from C 1s (C–S) → σ* (C–S) for the following reason. The intensity of peak III increases with decreasing *n* most noticeably for *n* from 3 to 1, which cannot be explained by the previous interpretation of this feature as a σ* (C–H)/Rydberg transition. If the latter were true, the intensity of this resonance would decrease with decreasing number of CH_2_ units (and possible σ* C–H and Rydberg transitions) in the backbone of the SAM. We therefore assign the resonance III to a transition involving the carbon directly bonded to the S-atom. The σ* (C–S) resonance has been reported to occur at a photon energy of 287.7 eV for phenylthiolates SAMs on Au and Mo substrates.^73–75^ The discrepancy in photon energy of ∼0.3 eV of peak III between the phenylthiolate and SC_*n*_Fc SAMs can be explained by the difference in the M–S bonds for SAMs derived from aromatic and aliphatic thiols.^76^

### Tilt angles of the Fc moieties

Angular dependent NEXAFS spectra (*cf.*Fig. 2) show that the intensities of the first two resonances I and II for SC_*n*_Fc on both Au and Au surface exhibit angular dependence over the entire range values of 1 ≤ *n* ≤ 15. For SC_*n*_Fc on Au, enhancement of resonances I and II is obtained at normal incidence (*θ* = 90°) for *n* = odd and at grazing incidence (*θ* = 20°) for *n* = even, whereas the reverse trend is observed for SC_*n*_Fc on Ag. The average tilt angles of a molecular unit can be quantified from the evolution of resonances as a function of incident angles measured using angular-dependent NEXAFS spectroscopy.^77^ The resonance intensity is enhanced when the electric field vector of the synchrotron light is parallel to the direction of the related molecular orbital defined by their maximum orbital wavefunction amplitude. For SC_*n*_Fc molecules, the π* orbital are orientated parallel to the Cp–Fe–Cp axis. Therefore, the average tilt angle (*α*) of the Fc moiety, defined as the angle between the Cp–Fe–Cp axis and surface normal (as indicated in Fig. 1), can be determined using the angular dependent NEXAFS spectra acquired at normal (90°) and grazing (20°) incidence. Assuming a random azimuthal orientation between molecules and substrate, *α* can be extracted from the intensity ratio of π* resonances (*I*_π*_) at 90° and 20° incident angles (*θ*) using eqn (1)^77^1
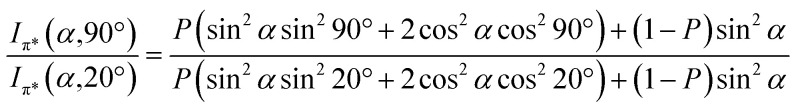
where *P* = 0.90 is the linear polarization factor of incident X-ray light. The intensity ratio peak I was used to estimate *α*.

**Fig. 3:**
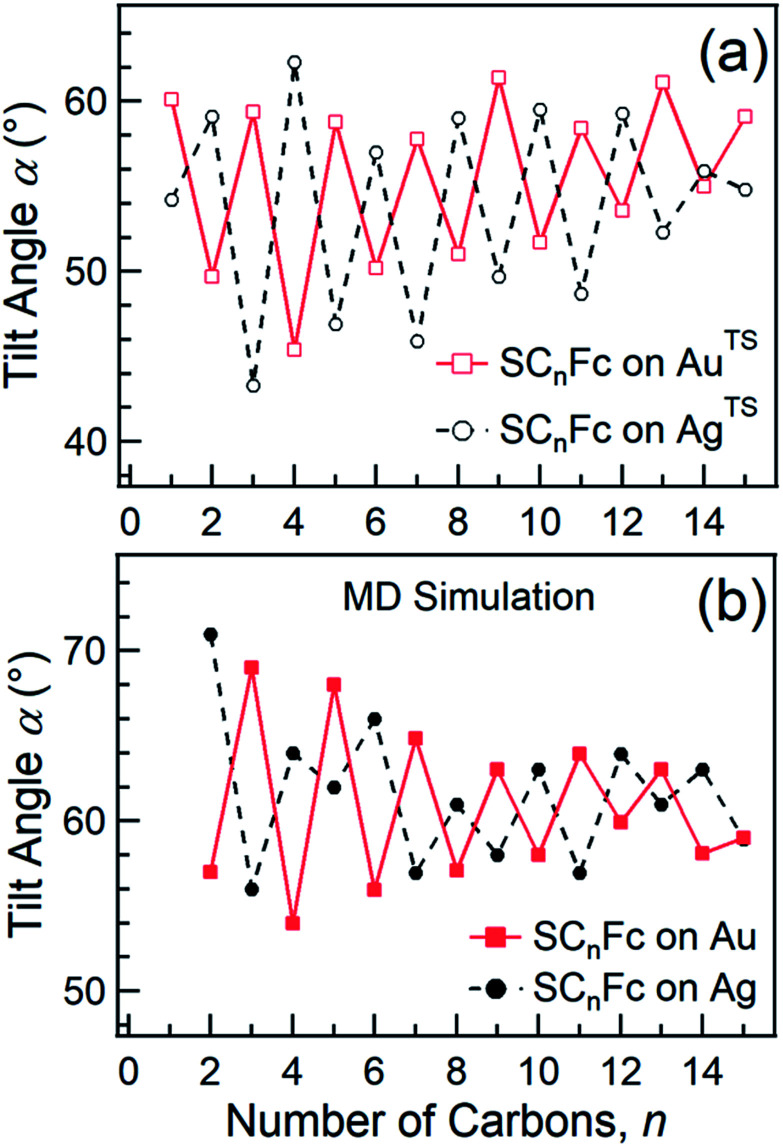
(a) Average tilt angles (*α*) of Fc moieties (with a relative error of roughly ±1° due to the uncertainty in the intensity of 5% of the signal of peak I) as a function of *n* for SC_*n*_Fc SAMs on Au^TS^ (open square) and Ag^TS^ (open circle) evaluated experimentally by angular dependent NEXAFS. Data for *n* = 1–5 for SAMs on Au are taken from ref. 55. Data for *n* = 6–15 for SAMs on Au are taken from ref. 19. Data for *n* = 6–15 for SAMs on Ag are taken from ref. 54. (b) The tilt angles of Fc moieties calculated by MD simulation (with a standard error of 5°) as a function of *n* for SC_*n*_Fc on Au (solid square) and Ag (solid circle). Data for *n* = 8–13 for SC_*n*_Fc on Au were taken from ref. 19, and *n* = 2–7 and *n* = 14, 15 for SC_*n*_Fc SAMs on Au were taken from ref. 55, respectively. Data for *n* = 6–15 for SC_*n*_Fc on Ag were taken from ref. 54.

In principle, NEXAFS spectra exhibit no angular dependence when the adsorbate molecules are oriented randomly or at the magic angle of 54.7°.^77^ Distinguishing between these two scenarios requires careful analysis and additional evidence. Fig. 3(a) shows *α* determined from our NEXAFS data using eqn (1) as a function of *n*. The odd–even effect is persistent for *n* = 1–15 for SAMs on both Au^TS^ and on Ag^TS^, but the odd–even effect in *α* is exactly opposite for the two substrates. We note that uncertainty in the absolute value of *α* of ±5° is caused by uncertainty of degree of polarization of the X-ray beam and angular misalignment due to sample mounting.^78^ These errors are expected to contribute equally to all samples, and therefore can be regarded as a systematic error. The relative error in *α vs. n* is ±1°, which is induced by the uncertainty in the intensity of 5% of the signal of peak I. Consequently, the odd–even effect observed in *α*, albeit small, reflects an intrinsic property of the supramolecular organization of the SC_*n*_Fc SAMs.

Fig. 3(b) shows the calculated tilt angles of the Fc moieties obtained by molecular dynamics (MD) simulations (see calculation details and full calculation dataset in ref. 55), which reproduce the odd–even and the reversal of the odd–even effect in *α*. The offset in the calculated and measured values could be ascribed to the systematic error associated with our beamline as mentioned above and/or in the force field describing the depth of the potential energy well associated with the metal–molecule bond angles and dihedral angles. This reversal of the odd–even effect is caused by the different C–S-metal binding geometries with C–S–Ag bond angle of ∼180°, and C–S–Au bond angle of ∼109° as indicated in Fig. 1 in agreement with earlier studies.^53,79–81^

### SAM thickness and surface coverage

The SAM thickness (*d*_SAM_ in Å), location of the Fe atom of the Fc within the SAM with respect to vacuum (*d*_Fe_ in Å), S-vacuum distance (*d*_S_ in Å; *cf.*Fig. 1(c)) and the surface coverages (*Γ* in mol cm^−2^), quantify the structural quality of SAMs on Au^TS^. The values of *d*_SAM_, *d*_Fe_, and *Γ*, were determined by angle-dependent PES following the procedure reported in ref. 54.

**Fig. 4:**
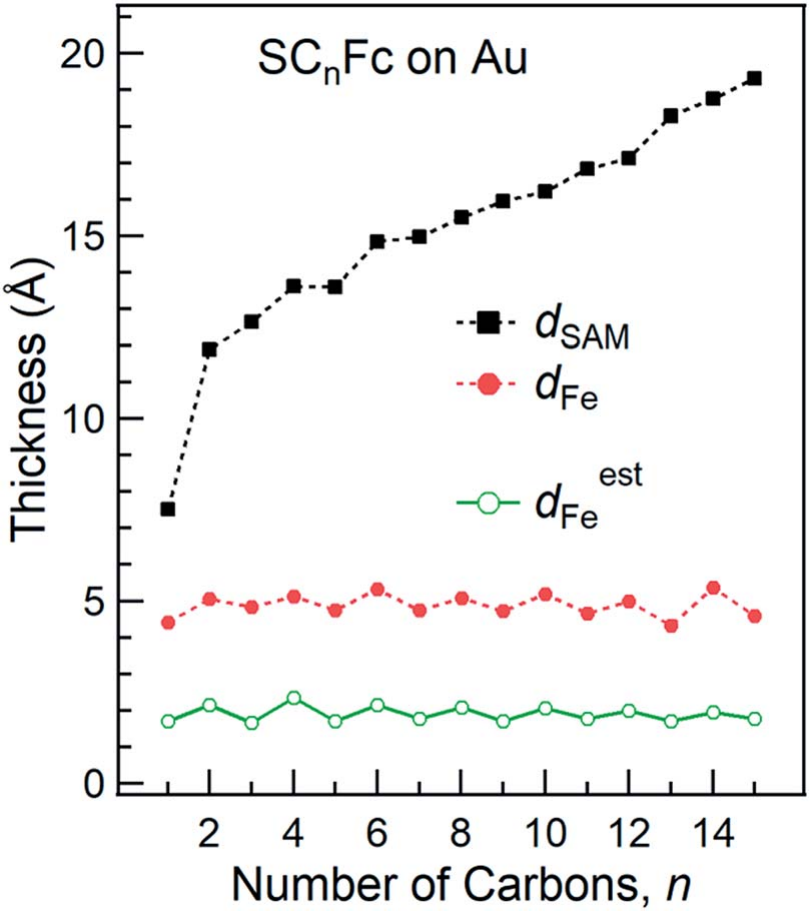
Thickness of SAMs and Fe-vacuum distance (with an error of 2 Å) calculated using angular dependent PES. Green line represents the estimated Fe-vacuum distances (*d*^est^_Fe_). The values of *d*_SAM_ were taken from ref. 55.

Fig. 4 shows how the values of *d*_SAM_ and *d*_Fe_ evolve as a function of *n*. The value of *d*_SAM_ increases with increasing *n*, while *d*_Fe_ is nearly independent of *n* with an average value of ∼4.9 Å. From these observations we conclude that the Fc moieties are mostly located at the top of the SAMs without significant back bending. Fig. 4 also shows a small odd–even effect as *d*_Fe_ is larger for *n* = even than for *n* = odd, which is consistent with the trend one would expect from the odd–even effect of *α* where the Fc moieties stand up more for *n*_even_ SAMs on gold than for *n*_odd_ SAMs resulting in slightly larger *d*_Fe_ values for *n*_even_. Considering *α* derived from NEXAFS (*cf.*Fig. 3) and using a Fe–Cp distance *d*_Fe–Cp_ = 3.35 Å (half the Fc length of 6.70 Å (ref. 82–84)), Fe-vacuum distances (*d*^est^_Fe_) for the entire series can be estimated by using eqn (2); these values are also plotted in Fig. 4 (green line). The estimated odd–even trend agrees well with experimental data as expected, although a constant offset is present likely due to error introduced by uncertainties in the values of inelastic mean free path (*λ*) when *d*_Fe_ is estimated using eqn (S2) (see ESI† for details).

2 *d*^est^_Fe_ = *d*_Fe–Cp_ × cos(*α*)

**Fig. 5:**
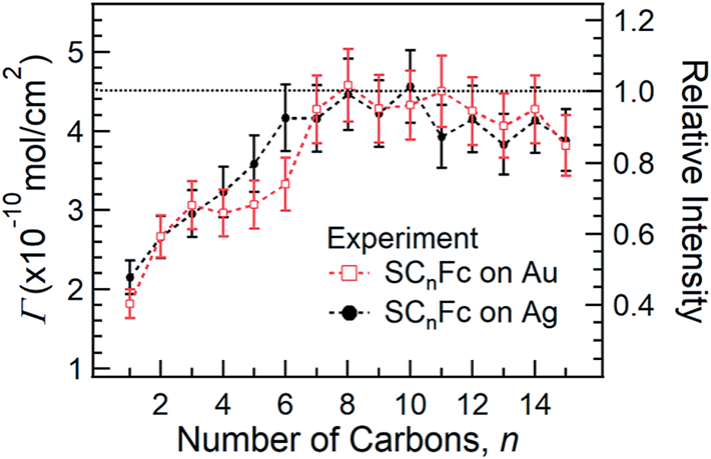
Surface coverage as a function of *n* for SC_*n*_Fc SAMs on both Au^TS^ (open square circle) and Ag^TS^ (solid circle) determined by Fe 2p_3/2_ spectra. The error bars represent maximum fitting error of 10%.

Fig. 5 shows the surface coverage *Γ* derived from the integrated intensity of the Fe 2p_3/2_ spectra as a function of *n* (*cf.* Fig. S2 and S3†). The reported value of *Γ* of 4.5 × 10^−10^ mol cm^−2^ for SC_11_Fc on Au serves as the reference value for the calculation.^82–84^ The value of *Γ* can be calculated by comparing the Fe 2p_3/2_ relative intensities for the two series with the intensity for SC_11_Fc on Au SAMs. The evolution of *Γ* as a function of *n* is almost identical for SAMs on Au and Ag. For *n* ≥ 7, *Γ* is nearly constant, but for *n* < 7 the value of *Γ* decreases substantially as the shorter SAMs form a more loosely packed SAM structure. In principle, the packing structures of the SAMs are determined by the balance of interactions between the molecule–substrate interactions and intermolecular interactions. As reported previously, the molecular backbone alkyl–alkyl interactions start to dominate for *n* ≥ 7, resulting in the densely packed structure associated with the standing up phase, whereas for SAMs with *n* < 7 Fc–Fc and Fc–alkyl van der Waals interactions dominate over the alkyl–alkyl interactions which can explain the loose packing of these SAMs.^55^ The reduced surface coverage does not strongly affect the average tilt angle of Fc moieties revealed by NEXAFS measurement (*cf.*Fig. 3) and the orientation of alkyl chains as, judged by the SAM thickness (*cf.*Fig. 4), from which we deduce that the lying-down phase is not present.

### Electronic structures

**Fig. 6:**
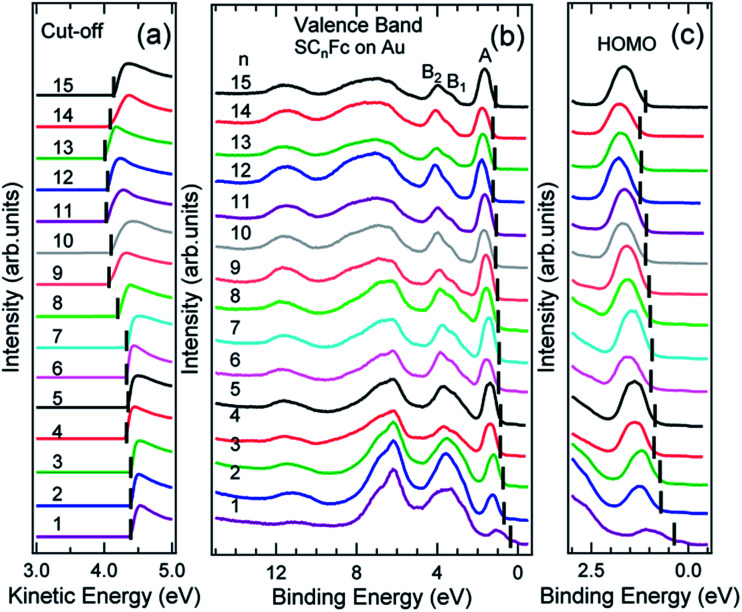
(a) The low kinetic energy secondary electron cut-off, (b) valence band, and (c) fine structures close to Fermi level for SC_*n*_Fc series SAMs on Au^TS^. The solid bars in panel (a) indicate the cut-off positions, whereas solid bars in panel (b) and (c) indicate the HOMO onset positions. Valence band spectra in panel (c) were taken from ref. 55. Data for *n* = 6–15 in panels (a) and (b) were taken from the ESI of ref. 54. All the data are shown here together for the sake of clarity.

**Fig. 7:**
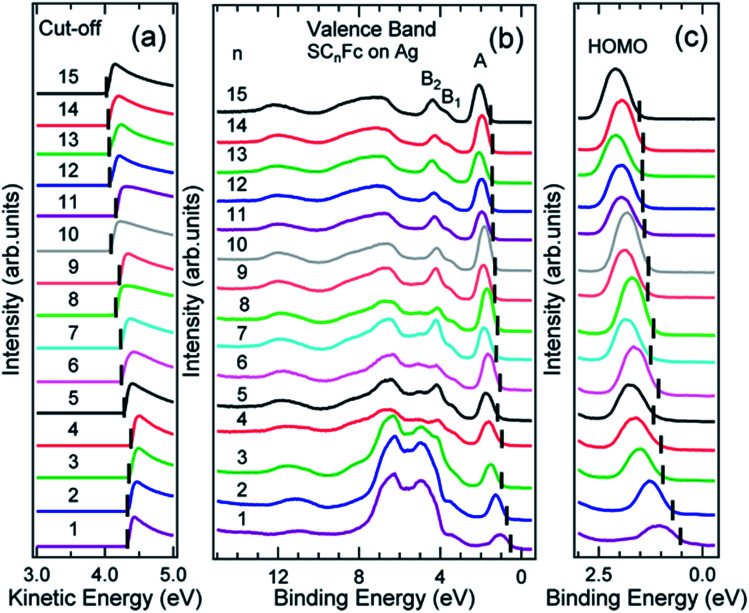
(a) Cut-off, (b) valence band, and (c) fine structures close to Fermi level for SC_*n*_Fc series SAMs on Ag^TS^. The solid bars in (a) and (b, c) present the cut-off and HOMO onset positions, respectively. Data for *n* = 6–15 in figure panel (a) and (b) were taken from the ESI of ref. 54. All the data are shown here together for the sake of clarity.

**Fig. 8:**
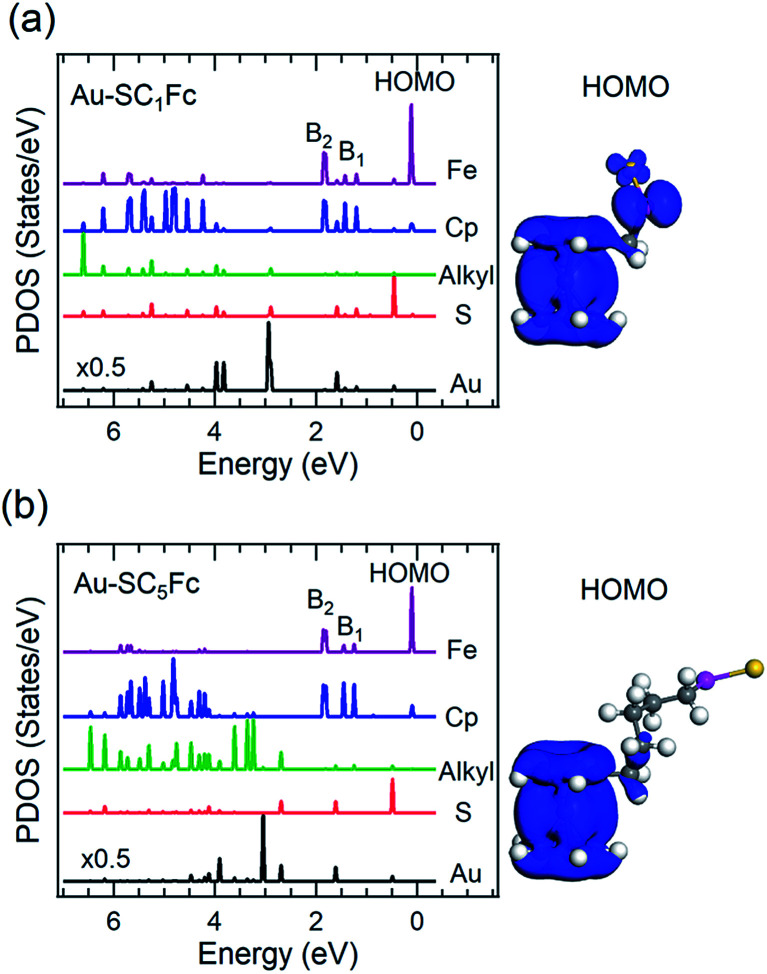
Calculated PDOS of Cp, Fe, alkyl, sulfur and Au for (a) Au–SC_1_Fc and (b) Au–SC_5_Fc molecules. HOMO orbitals of each molecule are displayed in the righthand panel.

Fig. 6 and 7 show the secondary electron cut-off (SECO), valence band spectra, and HOMO features close to *E*_F_ for SC_*n*_Fc SAMs on Au^TS^ and on Ag^TS^, respectively. To identify the molecular orbitals, we calculated the projected density of states (PDOS) of Au–SC_1_Fc and Au–SC_5_Fc using density functional theory (DFT) calculations, which are shown in Fig. 8. In Fig. 6(b) and 7(b), the feature close to the Fermi level in the region 0.5–2.5 eV (feature A) is mainly attributed to the Fc HOMO and is dominated by Fe 3d_*x*^2^–*y*^2^_ and 3d_*xy*_ orbitals hybridized with a minor contribution from Cp π orbitals revealed by the DFT results (*cf.*Fig. 8).^62,85^ Valence band features labeled as B_1_ and B_2_ are also attributed to Fc moieties and peak B_1_ is dominated by the π orbitals of Cp ring, whereas B_2_ mainly consists of the π bonding of the Cp ring hybridized with Fe 3d orbitals (*cf.*Fig. 8).^62^ It is worthwhile to note that the photon energy of 60 eV used to probe the valence band structures is close to the Fe 3p → 3d transition energy of ∼55 eV.^86^ This induces resonant enhancement of photoemission signals for those molecular orbitals that contain Fe 3d character,^87^ leading to the relatively high signal intensities of the HOMO and B_2_ peaks in our experimental spectra compared to other studies that used lower photon energies of *e.g.* 21.2 eV.^85,88^ Features observed in a binding energy range between 4.5 and 9.0 eV likely originate from σ (C–C) orbitals from both Fc moieties and the alkyl chains. It should be noted that for shorter SAMs (*n* < 7), the contribution of the substrate valence band features (*i.e.*, Au 5d ranging from 3 to 8 eV or Ag 4d ranging from 4 to 8 eV) are visible and they overlap with valence band features of the molecules above the HOMO peak.

### HOMO onset positions

**Fig. 9:**
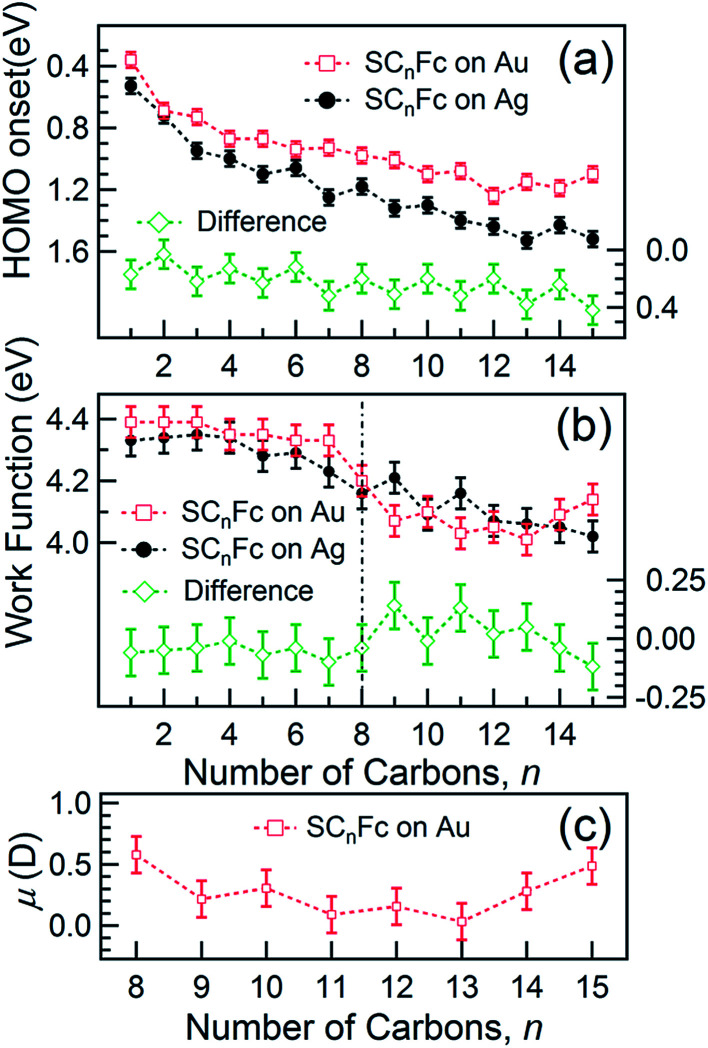
(a) HOMO onset position and (b) WF (with an error of 0.05 eV) as a function of *n* for SC_*n*_Fc series on Au^TS^ (open square) and Ag^TS^ (solid circle). The different spectrum is shown in the bottom of each panel. Date for *n* = 6–15 in panel (a) were taken from ref. 54. Data for SAMs on Au and *n* = 6–15 for SAMs on Ag in panel (b) were taken from ref. 55 and ref. 54, respectively. (c) The calculated *μ* for SC_*n*_Fc on Au^TS^ with *n* = 8–15 using eqn (3).

The *E*_HOMO_ in Fig. 9(a) generally shifts to higher binding energies with increasing values of *n* on both types of substrates, and remains relatively constant for *n* > 12. This observation can be explained by the photo-hole screening effect, in which the photo-hole in the molecular HOMO state is more effectively screened by the electronic relaxation of the metallic substrate. This screening is stronger when the Fc unit are close to the substrate, than when they are positioned far away, resulting in a lower BE for the HOMO.^89–92^ Hybridization between the Fc units and the metal substrate (*cf.* HOMO of Au–SC_1_Fc shown in Fig. 8(a)) results in broadening of the HOMO (*cf.* HOMO of Au–SC_1_Fc shown in Fig. 6(c)) and shifts *E*_HOMO_ to higher binding energies.

*E*_HOMO_ exhibits noticeable and opposite odd–even effects for SAMs on Au and Ag. Considering that the dielectric properties of Au and Ag are similar, we expect photohole screening on Au and Ag to be similar.^90^ Therefore, the difference in the *E*_HOMO_ between SAMs on Au and Ag, shown at the bottom of Fig. 9(a), helps to establish the opposite odd–even effect on the two substrates. We believe the weak modulation of the *E*_HOMO_ positions as *n* changes can be related to the odd–even effect in the orientation of the Fc units and the packing of the SAM, which, in turn, change the electronic polarization strength of the surrounding Fc moieties and, consequently, result in more efficient photohole screening (lower BE) for SAMs with the Fc units tilted more in parallel with the substrate (even *n* on Ag and odd *n* on Au) than when the Fc units are standing more upright.

### Work function

Fig. 9(b) shows the work function (WF) of the SAM-coated electrode, *ϕ*_SAM_, as a function of *n*. The WF changes upon molecular adsorption due to the formation of surface or interface dipoles which can be described using the Helmholtz equation^93–95^3
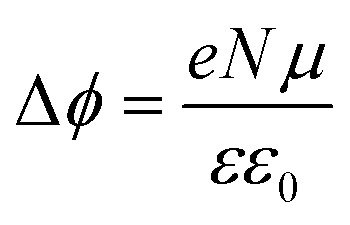
where *e* is the elementary charge which is 1.602 × 10^−18^ C, *N* is the dipole density which corresponds to the surface density of SAM molecules (molecule per m^2^) in this case (*cf.*Fig. 5), *μ* is net dipole moment projected along surface normal, *ε* is the relative dielectric constant which is typically 3 for organic molecules and *ε*_0_ is the vacuum permittivity of 8.85 × 10^−12^ F m^−1^.^96^

It is well-known that the change in the work function, Δ*ϕ*, of the bare metal substrate, due to molecular adsorption reflects the change in the surface potential through the formation of interface dipoles (IDs) composed of the bond dipole, *μ*_bond_, resulting from charge redistribution induced by electronic interactions between molecular adsorbates and metal substrates, and intrinsic molecular dipoles of the SAM molecules projected along surface normal, *μ*_mol_.^13,44,97–99^ Therefore, the molecules, once adsorbed on the metal surface, induce a change in work function of metal surface Δ*ϕ* which can be expressed as:4
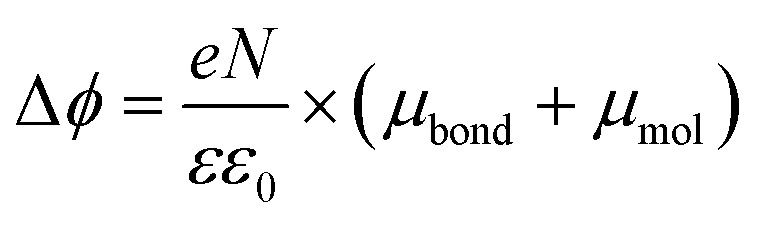


The decrease of WF by *μ*_bond_ is typically on the order of 1.4 to 0.4 eV for alkanethiolate SAMs on Au or Ag.^13,100^ The effect of the molecular dipoles (*μ*_mol_) on the metal work function depends not only on the individual molecular structures of the SAMs, but also on the collective order and orientation of the SAMs, as the latter directly affects the surface normal projection of the SAM dipoles. Here, the major contribution to *μ*_mol_ is the projected dipole moment along the surface normal resulting from the net dipole moment pointing towards the alkyl chain (*i.e.*, away from vacuum; *cf.*Fig. 1) which is connected to the negatively charged Cp resulting in an asymmetrically substituted Fc moiety. This dipole increases the surface work function. The interfacial interaction between the metal and the sulphur atom has significant influence on the value of *μ*_bond_. In the following sections, we will discuss in detail how *μ*_mol_ and *μ*_bond_ depend on *n*.

#### Odd–even effect for *n* ≥ 8

For long alkyl chains of *n* ≥ 8, on one hand, the values of *μ*_bond_ are expected to be similar for all SAMs^13^ since the Fc units and the metal substrate are electronically decoupled. On the other hand, alkyl packing dominates over Fc packing as mentioned above. As a result, the molecular backbones pack well and form well-ordered SAMs (in agreement with the surface coverage shown in Fig. 5). In this regime, the variation of work function SAM (Δ*ϕ*_SAM_) is then associated only with the change in the Fc-induced intramolecular dipole term (*μ*_mol_) and can be expressed as:5
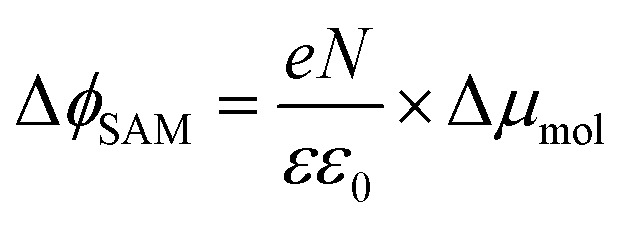


In other words, it is expected to observe an odd–even effect in the WF for SAMs with *n* ≥ 8 caused by the odd–even variation of the orientation of the molecular dipole. Note that earlier DFT models showed similar magnitude work functions and decrease with increasing *n* but did not show the odd–even effect due to the lack of dynamics in the zero Kelvin electronic structure models.^17^ Considering a constant dipole moment along the Fc–(CH_2_)_*n*_ axis for all Fc–SAMs studied here, its contribution along the surface normal is expected to exhibit the same odd–even effect as the Fc orientation.^54^ As illustrated in Fig. 1(c) and (f) for SAMs on Au or Ag with *n* = 8 and *n* = 9, the resulting projected *μ*_mol_ is lower for SAMs with the more in-plane tilted Fc moieties (*i.e.*, *n* = odd on Au and *n* = even on Ag) than those SAMs with more upright Fc units orientated along the surface normal (*i.e.*, *n* = even on Au and *n* = odd on Ag). Considering that *μ*_mol_ here increases the surface work function, it is therefore expected that WF of SAMs with more lying-down Fc moieties is lower than that of SAMs with the Fc units standing more up-right. This is clearly consistent with the experimental measurements of WF as shown in Fig. 9(b) (as well as the difference profile shown at the bottom) showing a clear odd–even effect which is reversed on Au and Ag for *n* ≥ 8.

To estimate the work function change solely due to *μ*_mol_ for SC_*n*_Fc SAMs on Au^TS^ with *n* = 8–15, the reference WF of 4.0 eV for SC_12_ SAMs on Au^TS^ is used, which also includes the contributions from *μ*_bond_,^13^ and *μ*_mol_ can be calculated using eqn (5). Fig. 9(c) shows a clear odd–even effect in *μ*_mol_. The average difference between *n* = even and *n* = odd in the dipole moment is estimated to be Δ*μ*_mol_ = 0.12*D* or 0.40 × 10^−30^ C m (in Debye, 1 *D* = 3.336 × 10^−30^ C m), which is an order of magnitude larger than the estimated ∼0.01 *D* between the odd and even *n*-alkanethiols on Au because of larger molecular dipole located at Fc terminal moieties in our case.^101^

#### Changes in WF for 3 ≤ *n* ≤ 7

The odd–even effect is quenched for *n* < 8, and an apparent increase in WF occurring around the transition region of *n* = 8 can also be observed; this can be attributed to an increase in the coupling strength between Fc units and the substrate for shorter SAMs *via* van der Waals interactions^17^ and the decrease of the surface coverage. Van der Waals interactions reduce molecular dipole *μ*_mol_ (*cf.*Fig. 1(b) and (e)) and, consequently, reduce the odd–even effect in WF. In the intermediate region of 3 ≤ *n* ≤ 7, the molecular backbones are loosely packed (*cf.*Fig. 5) because of weak interactions between the alkyl chains. The SAM packing energy is dominated by Fc–Fc interactions, resulting in a lower packing density and, thus, a lower dipole density for *μ*_bond_. Hence, we measure a higher WF than for SAMs in the more densely packed regime (*i.e. n* ≥ 8).

#### Changes in WF for *n* < 3

For *n* < 3, hybridization between Fc and metal substrate through S-atoms occurs as evidenced by the DFT calculations shown in Fig. 8(b), corresponding well with previously reported results.^17,62^ However, the WF is still significantly lower than that of bare metals owing to the formation of bond dipoles through the metal–Fc hybridization (*μ*_bond_, *cf.*Fig. 1(a) and (d)).^17^ We thus propose that an accumulation of electrons close to the metal surface, as a result of hybridization, yields a dipole outwards to vacuum.

We surmise that the WF values of ordered SAMs can be fine-tuned by controlling the orientation of terminal moieties of SAMs (through precisely controlling *n*) when their coupling strength with the substrate is weak. Together with the position of the HOMO onsets, WF shifts could have a deterministic effect on the charge transport inside the SAMs. Consequently, understanding the electronic structures, including the position of HOMO onset and the magnitude of the WF shift, and their dependence on the orientation of terminal groups, chain lengths, and packing structures, have important implications for the rational design of SAM-based devices.

## Conclusions

The supramolecular structures and electronic structures for a series of SC_*n*_Fc (1 ≤ *n* ≤ 15) SAMs on Au^TS^ and Ag^TS^ have been systemically investigated using synchrotron-based PES and NEXAFS, DFT, and MD simulations, from which we make the following observations: (i) the average tilt angles of the Fc moieties show significant and opposite odd–even effects on Au and Ag surfaces due to different C–S–metal angles, (ii) the thickness of the SAMs increases linearly with increasing *n*, and (iii) the SAM surface coverages are more ideal for *n* ≥ 8 than for shorter SAMs, which can be attributed to competition between Fc and alkyl packing with shorter chains.^19,54,55^

The electronic structures are strongly affected by the supramolecular SAM structure which determines the Fc orientation and coupling strength between the Fc units and the substrate. The HOMO onset positions follow the odd–even effect in the orientation of the Fc moieties for all values of *n*, which raises the possibility of using SAMs to fine-tune charge injection barriers in organic electronic devices. The coupling strength between the Fc units and the substrate plays an important role in the WF of the system on both types of substrates. In the region of *n* ≥ 8, the Fc units are decoupled from the substrate by the long alkyl chains. Here, the alkyl chain interactions drive the SAM packing and the intrinsic surface dipoles in the ordered SAM result in an odd–even effect in WF that follows the odd–even trend in the Fc orientation. The odd–even effect in WF is quenched for 3 ≤ *n* ≤ 7 even though the odd–even effect in the HOMO onset values persists. This can be explained by the van der Waals interactions between the Fc units and the substrate. In this regime, the SAM packing interactions are weaker than for SAMs with *n* ≥ 8 which results in reduced surface coverages and in an increase of the WF. For SAMs with *n* < 3, hybridization between the Fc moieties and the metal surface dominates the electronic structure of the system.

Our results show that small changes in the supramolecular structure and molecular orientation of SAMs can induce significant changes in the electronic structure of the SAM–electrode interface. The model system we studied here is a molecular diode for which the device performance is very sensitive to minute changes in supramolecular and electronic structure. Our detailed investigation into the molecular orientation, supramolecular structures and interfacial electronic structures at SC_*n*_Fc SAM/metal interfaces can help us improve our understanding of the Fc–SAM system and relate the structural and electronic properties to the electron transport measurements, hence providing design rules to optimize the device properties and to develop novel applications. Thus the function of molecular–electronic devices, and in more general (bio)organic electronics, can only be understood once the electronic and supramolecular structures, and how they influence each other, are understood.

## Conflicts of interest

There are no conflicts to declare.

## Supplementary Material

NA-001-C9NA00107G-s001
