# Supplementary material for: The supramolecular structure and van der Waals interactions affect the electronic structure of ferrocenyl-alkanethiolate SAMs on gold and silver electrodes
Source: Nanoscale Adv. 2019 Mar 29;1(5):1991–2002. doi: 10.1039/c9na00107g (PMC9417838; doi:10.1039/c9na00107g)
Supplement: NA-001-C9NA00107G-s001 [file NA-001-C9NA00107G-s001.pdf]

Electronic Supplementary Material (ESI) for Nanoscale Advances.
This journal is © The Royal Society of Chemistry 2019

Supplemental Information

The Supramolecular Structure Affects the Electronic Structure of Ferrocenyl-Alkanethiolate SAMs on Gold and Silver Electrodes

Liang Cao,[1,2,3#] Li Yuan,[1#] Ming Yang,[4] Nisachol Nerngchamnong,[1] Damien Thompson[5] Yu Xiaojiang,[6] Dong-Chen Qi,[7,8] and Christian A. Nijhuis[1,9,10*]*

1. Department of Chemistry, National University of Singapore, 3 Science Drive 3, Singapore 117543, Singapore

2. Anhui Province Key Laboratory of Condensed Matter Physics at Extreme Conditions, High Magnetic Field Laboratory of the Chinese Academy of Sciences, 350 Shushanhu Road, Hefei 230031, China

3. Department of Physics, National University of Singapore, 2 Science Drive 3, Singapore 117542, Singapore

4. Institute of Materials Research and Engineering (IMRE) 2 Fusionopolis Way, Innovis, Singapore 138634, Singapore

5. Department of Physics, Bernal Institute, University of Limerick, V94 T9PX, Ireland

6. Singapore Synchrotron Light Source, National University of Singapore, 5 Research Link, Singapore 117603, Singapore

7. School of Chemistry, Physics and Mechanical Engineering, Queensland University of Technology, Brisbane, Queensland 4001, Australia

8. Department of Chemistry and Physics, La Trobe Institute for Molecular Science, La Trobe University, Melbourne, Victoria 3086, Australia

9. Centre for Advanced 2D Materials and Graphene Research Centre, National University of Singapore, 6 Science Drive 2, Singapore 117546, Singapore

10. NUSNNI-Nanocore, National University of Singapore, Singapore 117411, Singapore

*Authors to whom correspondence should be addressed: dongchen.qi@qut.edu.au (D.-C. Qi) and chmnca@nus.edu.sg (C. A. Nijhuis)

# Both authors contributed equally to this work.

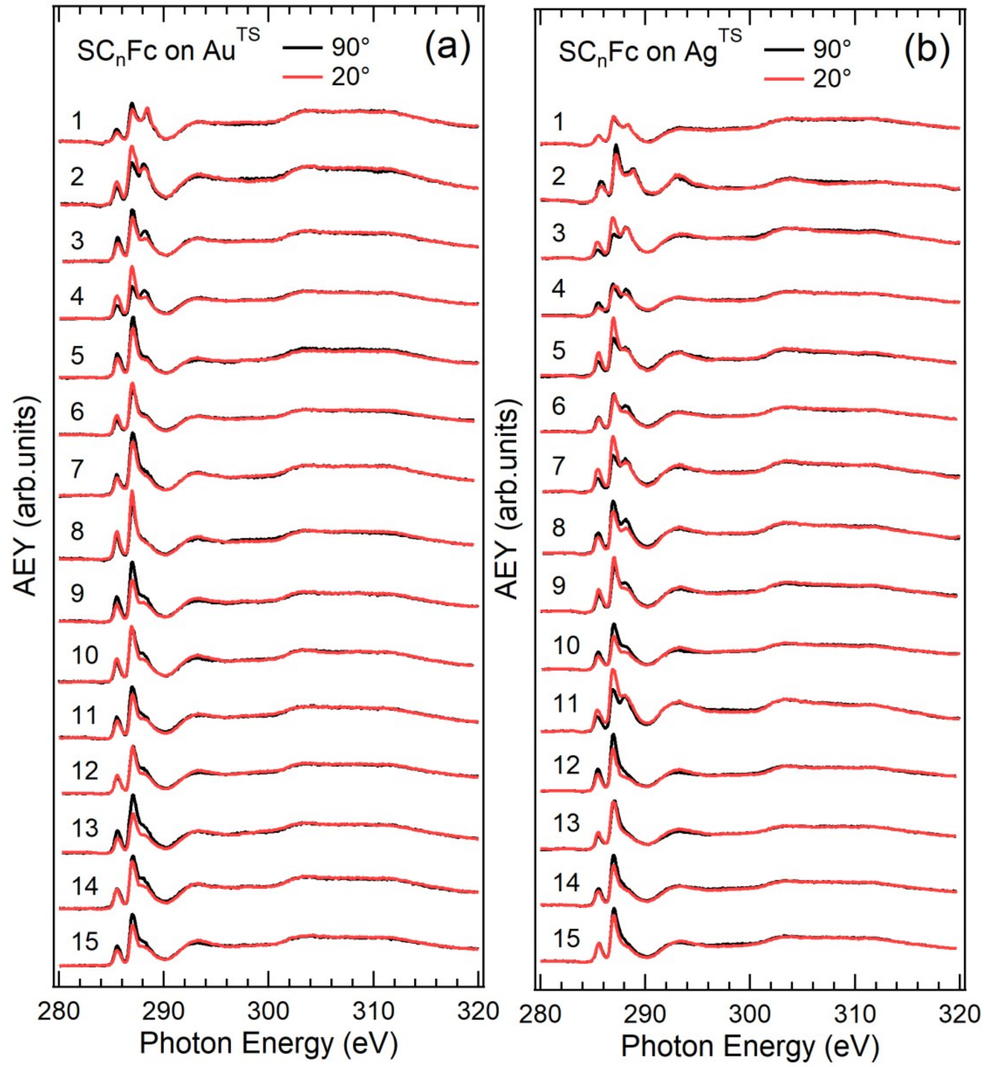

Figure S1. NEXAFS spectra for SC_nFC SAMs on Au^{TS} (a) and on Ag^{TS} (b). Data for $n = 3$ and 4 for SAMs on Au were taken from ref[1]. Data for $n = 6$-15 were taken from supporting information of ref[2].

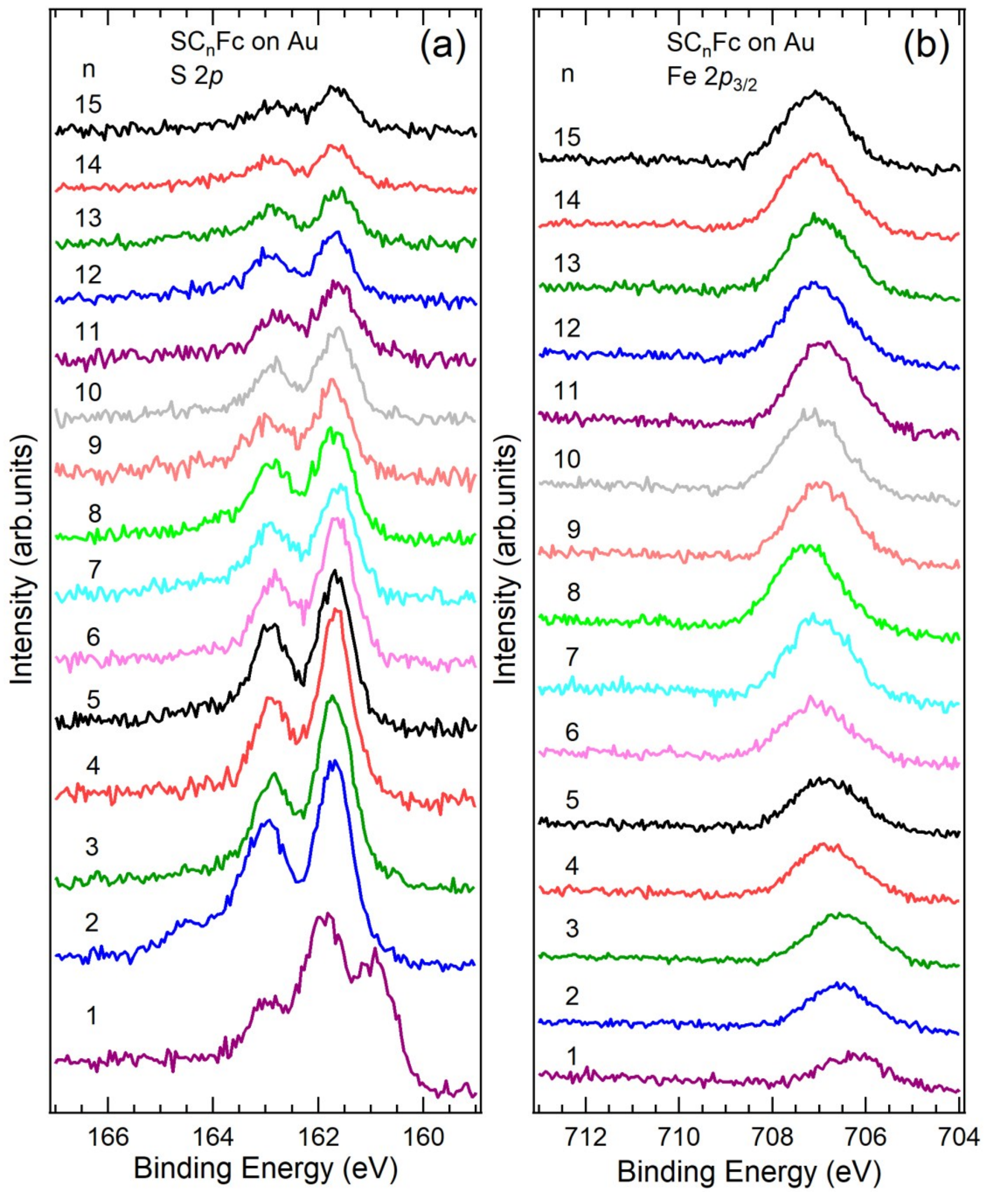

Figure S2. S 2*p* (a) and Fe 2$p_{3/2}$ spectra for SC_nFc on Au^{TS} measured normal emission angle. The intensity of the S 2*p* component that is exclusively associated with the S-Au bonds (~161.7 eV) was used to evaluate the d_{SAM}. S 2*p* spectra were taken from supporting information of ref[3]. Fe 2$p_{3/2}$ spectra for n = 6-15 were taken from supporting information of ref[4]. All data are shown together for the sake of clarity.

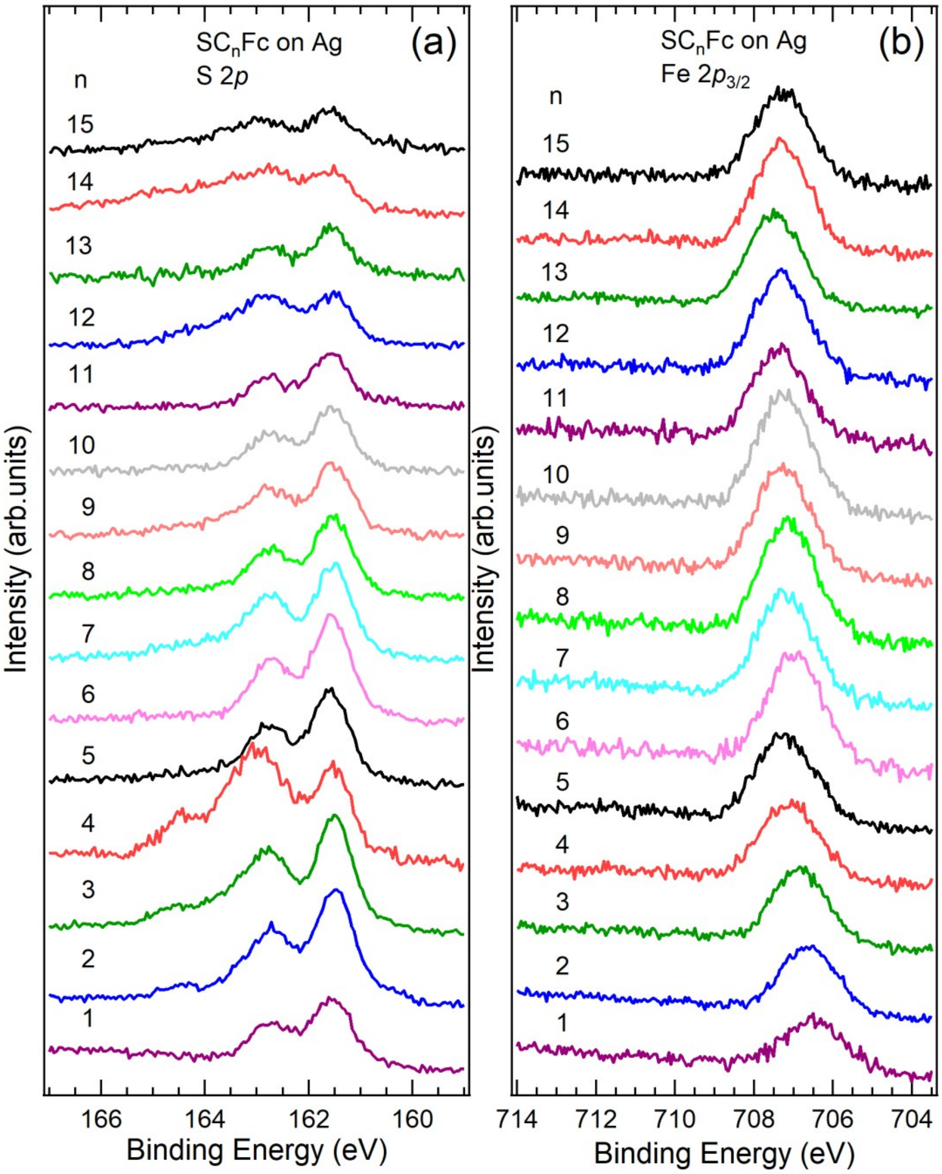

Figure S3. S 2p (a) and Fe 2$p_{3/2}$ spectra for SC$_n$Fc on Ag^{TS} measured normal emission angle. S 2p and Fe 2$p_{3/2}$ spectra for n = 6-15 were taken from supporting information of ref[4]. All data are shown together for the sake of clarity.

Table S1: Relative intensities (I_{eff}) at 90º and 40º take-off angles for S 2*p* and Fe 2$p_{3/2}$ components of SC_nFc SAMs on Au^{TS}. The d_S, d_{Fe}, and d_{SAM} are evaluated from angular dependent PES following the procedure reported in ref [4].[a] Average tilt angles of Fc (α) are estimated from angular dependent NEXAFS.

n	S 2*p*			Fe 2$p_{3/2}$			d_{SAM} (Å)	α(°)
	I_{eff}(90º) (%)	I_{eff}(40º) (%)	d_S (Å)	I_{eff}(90º) (%)	I_{eff}(40º) (%)	d_{Fe} (Å)		
1	61.0	39.0	5.72	61.3	38.7	4.43	7.52	59.2
2	68.0	32.0	10.13	62.5	37.5	5.07	11.93	49.9
3	69.1	30.9	10.87	62.1	37.9	4.86	12.67	60.2
4	70.5	29.5	11.82	62.6	37.4	5.12	13.62	45.4
5	70.5	29.5	11.82	61.9	38.1	4.75	13.62	59.3
6	72.2	27.8	13.02	63.0	37.0	5.34	14.82	50.0
7	72.4	27.6	13.16	62.0	38.0	4.80	14.96	58.1
8	73.2	26.8	13.74	62.6	37.4	5.12	15.54	51.5
9	73.8	26.2	14.19	61.9	38.1	4.75	15.99	59.5
10	74.1	25.9	14.41	62.7	37.3	5.18	16.21	52.1
11	74.9	25.1	15.02	61.7	38.3	4.64	16.82	58.0
12	75.3	24.7	15.33	62.4	37.6	5.02	17.13	53.5
13	76.8	23.2	16.51	61.1	38.9	4.32	18.31	59.5
14	77.4	22.6	17.00	63.1	36.9	5.39	18.80	54.5
15	78.0	22.0	17.50	61.6	38.4	4.59	19.30	58.0

[a] Relative intensities of each component are evaluated with respect to the total intensity at two take-off angles.

References

1. N. Nerngchamnong, H. Wu, K. Sotthewes, L. Yuan, L. Cao, M. Roemer, J. Lu, K. P. Loh, C. Troadec, H. J. W. Zandvliet and C. A. Nijhuis, *Langmuir*, 2014, **30**, 13447-13455.
2. N. Nerngchamnong, L. Yuan, D.-C. Qi, J. Li, D. Thompson and C. A. Nijhuis, *Nat. Nano.*, 2013, **8**, 113-118.
3. N. Nerngchamnong, D. Thompson, L. Cao, L. Yuan, L. Jiang, M. Roemer and C. A. Nijhuis, *J. Phys. Chem. C*, 2015, **119**, 21978-21991.
4. L. Yuan, D. Thompson, L. Cao, N. Nerngchangnong and C. A. Nijhuis, *J. Phys. Chem. C*, 2015, **119**, 17910-17919.
